# CFAP70 Is a Novel Axoneme-Binding Protein That Localizes at the Base of the Outer Dynein Arm and Regulates Ciliary Motility

**DOI:** 10.3390/cells7090124

**Published:** 2018-08-29

**Authors:** Noritoshi Shamoto, Keishi Narita, Tomohiro Kubo, Toshiyuki Oda, Sen Takeda

**Affiliations:** 1 Department of Anatomy and Cell Biology, Interdisciplinary Graduate School, University of Yamanashi, Chuo, Yamanashi 409-3898, Japan; nori10shi.shamo10@gmail.com; 2 Department of Anatomy and Structural Biology, Interdisciplinary Graduate School, University of Yamanashi, Chuo, Yamanashi 409-3898, Japan; tkubo@yamanashi.ac.jp (T.K.); toda@yamanashi.ac.jp (T.O.)

**Keywords:** tetratricopeptide repeat containing protein, outer dynein arm, cryo-electron tomography

## Abstract

In the present study, we characterized CFAP70, a candidate of cilia-related protein in mice. As this protein has a cluster of tetratricopeptide repeat (TPR) domains like many components of the intraflagellar transport (IFT) complex, we investigated the domain functions of particular interest in ciliary targeting and/or localization. RT-PCR and immunohistochemistry of various mouse tissues demonstrated the association of CFAP70 with motile cilia and flagella. A stepwise extraction of proteins from swine tracheal cilia showed that CFAP70 bound tightly to the ciliary axoneme. Fluorescence microscopy of the cultured ependyma expressing fragments of CFAP70 demonstrated that the N-terminus rather than the C-terminus with the TPR domains was more important for the ciliary localization. When CFAP70 was knocked down in cultured mouse ependyma, reductions in cilia beating frequency were observed. Consistent with these observations, a *Chlamydomonas* mutant lacking the CFAP70 homolog, FAP70, showed defects in outer dynein arm (ODA) activity and a reduction in flagellar motility. Cryo-electron tomography revealed that the N-terminus of FAP70 resided stably at the base of the ODA. These results demonstrated that CFAP70 is a novel regulatory component of the ODA in motile cilia and flagella, and that the N-terminus is important for its ciliary localization.

## 1. Introduction

Motile cilia and flagella are highly conserved, filamentous subcellular organelles that propel extracellular fluids [1]. In vertebrates, defects in ciliary motility lead to many complications, such as infections in the respiratory tract, infertility, hydrocephalus, and congenital deformities, such as situs inversus [2]. They have a specialized cytoskeletal framework called the 9 + 2 axoneme, and the rhythmic, whip-like motion is generated as a result of the sliding movement between the outer doublets produced by the outer and inner dynein arms (ODAs and IDAs) [3,4,5,6] and various accessory structures, such as the nexin–dynein regulatory complex (N-DRC), radial spokes, and the central pair projection [7,8,9,10] (the atypical nodal cilia lack the central pair and other accessory structures and produce rotational movement [11]). These ciliary proteins are synthesized in the cytoplasm [12,13,14,15] and then transported along the axoneme by the intraflagellar transport (IFT) complex [16,17,18,19]. *Chlamydomonas reinhardtii* has long been used as an invaluable model organism to make milestone discoveries in the genomics, biochemistry, and biophysics of motile cilia and flagella [20,21,22]. With recent technical advances in cryo-electron tomography, the fine ultrastructure of the flagellar axoneme has been resolved at the molecular level [23]. However, while hundreds of flagellar proteins have been identified by previous proteomic analysis [24], which are termed systematically as flagella-associated proteins (FAPs), many of them remain uncharacterized.

In the present study, we characterize mouse CFAP70 (cilia- and flagella-associated protein 70; formerly known as tetratricopeptide-repeat-containing protein 18, TTC18) and the *Chlamydomonas* homolog FAP70. We focused on this molecule based on our transcriptome analysis in mice to seek novel cilia-related genes with unknown functions. Despite the name, experimental data demonstrating the association of CFAP70 with cilia have not been reported in mice. Little is known about this protein, except that it has tetratricopeptide repeat (TPR) domains that cluster at the C-terminus. TPR domains, each of which tends to form a pair of antiparallel alpha helices, are known to mediate protein–protein interactions, and are present in the majority of protein subunits of the IFT complex. Taschner et al. demonstrated the crystal structure of the IFT70/52 subcomplex, where the 15 tandem TPR domains of IFT70 adopt a superhelical structure to bind to the proline-rich motif of IFT52 [25]. Taking this into consideration, we hypothesized initially that CFAP70 might associate with IFT through the TPR domains. However, it turns out that CFAP70 binds to the axoneme in close proximity to the ODA independent of the TPR domains and regulates ODA activity. In this regard, this study may pave the way to the elucidation of subclinical ciliopathy in humans.

## 2. Materials and Methods

### 2.1. Antibodies

Rabbit anti-CFAP70 (HPA037582) and mouse anti-acetylated α-tubulin (6-11B-1) were from Sigma-Aldrich (St. Louis, MO, USA). Rabbit anti-IFT88 (13967-1-AP) was from Proteintech (Rosemont, IL, USA). Mouse anti-dynein intermediate chain (DNAIC1, GTX26304) was from GeneTex (Irvine, CA, USA). Rabbit anti-green fluorescent protein (GFP) (A-6455) and Alexa-Fluor-conjugated secondary antibodies were from Life Technologies (Carlsbad, CA, USA). Horseradish-peroxidase- and alkaline-phosphatase-conjugated secondary antibodies were from Cell Signaling Technologies (Danvers, MA, USA).

Rabbit polyclonal antibodies against *Chlamydomonas reinhardtii* FAP70 were produced using a synthetic oligopeptide SEGGVYQYSVVKHFC (Cosmo Bio, Tokyo, Japan) at EveBioscience (Wakayama, Japan). For affinity purification of anti-FAP70 antibodies, a GeneArt Strings DNA fragment corresponding to the coding sequence of FAP70 in exons 2 and 3 was synthesized by Thermo Fisher Scientific (Waltham, MA, USA) and ligated into pGEX6P-1 (GE Healthcare Japan, Tokyo, Japan). The purified recombinant FAP70 fragment was resolved by SDS-PAGE, transferred onto a PVDF membrane, and the membrane strip was used for affinity purification of the antibodies as described previously [26,27].

### 2.2. Animals and Cell Culture

Animal care was performed in accordance with institutional guidelines outlined by the Institutional Animal Care and Use Committee of the University of Yamanashi (Approval number: 19-92). Wild-type mice (C57Bl/6J) were produced in our institutes or purchased from Charles River Laboratories Japan (Yokohama, Japan). Primary culture of mouse ependyma was prepared as described previously [28]. Lenti-X 293T cells (Takara Bio, Shiga, Japan) were cultured following the manufacturers’ instructions.

### 2.3. Reverse Transcription-Polymerase Chain Reaction (RT-PCR)

Total RNA from various tissue and cell samples was extracted using Trizol Reagent (Life Technologies). Reverse transcription and the following semi-quantitative PCR were performed using High Capacity cDNA Reverse Transcription Kits (Applied Biosystems Japan, Tokyo, Japan) and KAPA HiFi DNA polymerase (KAPA Biosystems, Wilmington, MA, USA), respectively, following the manufacturers’ instructions.

### 2.4. Immunostaining

Mouse tissue sections on coated glass slides and cultured cells on glass covers were prepared as described previously [29]. The primary antibodies used for immunostaining and their dilutions were as follows: CFAP70 (1:500), acetylated α-tubulin (1:1000), IFT88 (1:500), GFP (1:200). The secondary antibodies used were Alexa Fluor 488-conjugated goat anti-mouse (1:200) and Alexa Fluor 568-conjugated goat anti-rabbit (1:200). As a negative control, normal immunoglobulins were used as the primary antibody. Confocal microscopy was performed with an Olympus FV10i-DOC laser-scanning microscope equipped with a built-in oil immersion objective equivalent to UPLSAPO 60XO (NA 1.35) at room temperature. The images were acquired with Fluoview software version 04.01 (Olympus, Tokyo, Japan) at 2× or 10× digital zoom. Overlaying images were made using ImageJ version 1.52a (National Institutes of Health, Bethesda, MD, USA).

### 2.5. Isolation and Subfractionation of Swine Tracheal Cilia

For the isolation of swine tracheal cilia, the soft layers of mucosa and submucosa were freshly dissected from the underlying cartilaginous layer, rinsed briefly with HBSS, and transferred to a 50-mL tube containing ice-cold HBSS at a local slaughterhouse. The tissue was then chopped into small pieces in a plastic dish containing the medium, transferred back to the same tube, and centrifuged at 500× *g* for 3 min to discard the supernatant. Then, the pellet was resuspended four times in 10 mL of buffer containing 10 mM Tris-HCl, pH 7.5, 50 mM NaCl, 10 mM CaCl_2_, 1 mM EDTA, 7 mM β-mercaptoethanol, and 1% protease inhibitor cocktail [30], vortexed for 1 min, and centrifuged at 500× *g* for 3 min at 4 °C to collect the supernatant containing liberated cilia. The pooled supernatant was again centrifuged at 500× *g* for 5 min at 4 °C to remove debris, and cilia were spun down at 8000× *g* for 10 min at 4 °C.

For subfractionation of cilia, the 8000× *g* pellet was first resuspended four times with 0.6 mL HMDEK (30 mM HEPES, pH 7.4, 5 mM MgSO_4_, 1 mM DTT, 0.5 mM EDTA, 25 mM KCl) containing 0.1% Triton X-100 and 1% protease inhibitor cocktail, incubated on ice for 2 min each, and centrifuged at 5000× *g* for 5 min at 4 °C to collect the supernatant. Next, the pellet was extracted twice with 1 mL HMDEK containing 0.6 M KCl and 1% protease inhibitor cocktail, incubated on ice for 15 min each, and centrifuged at 5000× *g* for 5 min at 4 °C to collect the supernatant. Finally, the pellet was extracted twice with 1 mL HMDEK containing 0.5 M KI and 1% protease inhibitor cocktail, incubated on ice for 30 min each, and centrifuged at 5000× *g* for 5 min at 4 °C to collect the supernatant.

The proteins in the three extracts were precipitated with 5% (*w*/*v*) TCA, rinsed three times with water, dissolved in 20 µL 4 × SDS sample buffer, and analyzed by Western blot.

### 2.6. Construction of Lentiviral Plasmid Expressing Full-Length and Truncated CFAP70

The cDNAs encoding the full-length *Cfap70* as well as the N- and C-terminal fragments were subcloned into a lentiviral GFP expression vector CS-CDF-CG-PRE (kindly provided by Dr. Hiroyuki Miyoshi at the RIKEN BioResource Center (Ibaraki, Japan), along with the packaging plasmids, pCAG-HIVgp and pCMV-VSV-G-RSV-Rev). The resulting constructs were designated as CFAP70-FL-GFP (full-length), CFAP70-N-GFP (the N-terminal fragment), or CFAP70-C-GFP (the C-terminal fragment).

### 2.7. Knockdown of CFAP70 Using Lentiviral shRNA and Live Imaging of Ependymal Cilia

For the knockdown of *Cfap70* expression in cultured mouse ependyma, the cultured mouse ependyma was transduced with lentiviral particles made with the lentiviral shRNA expression vector TRCN0000346338 (Dharmacon, Lafayette, CO, USA) at a multiplicity of infection of 1.0 when the culture reached confluency (around 12 days in vitro). As a negative control, an empty vector with a mock shRNA sequence (#RHS4080, Dharmacon) was used. Three days after viral transduction, 1.0 µg/mL puromycin was added to the culture medium for 5 days to eliminate untransduced cells.

For the analysis of cilia beat frequency, high-speed video microscopy was conducted by using an Olympus IX71 inverted microscope equipped with a 100 W mercury lamp, differential interference contrast optics, a UPLSApo 40X2 (NA 0.95) objective, and an Allied GE680 charge-coupled device (CCD) camera at room temperature as described previously [28]. The movies were acquired and analyzed with TI Workbench software version 12/12/2016 written by Dr. Takafumi Inoue (Waseda University, Tokyo, Japan).

### 2.8. CRISPR/Cas9-Mediated Knockout of fap70 in Chlamydomonas

*Chlamydomonas fap70-null* mutant was generated based on a previously reported method [31] with modifications. Ten microliters (10 µL) of 40 µM tracrRNA (5′-AAACAGCAUAGCAAGUUAAAAUAAGGCUAGUCCGUUAUCAACUUGAAAAAGUGGCACCGAGUCGGUGCU-3′) and 10 µL of 40 µM crRNA (5′-GAGCACCGAGGGUCUCGAUCGUUUUAGAGCUAUGCUGUUUUG-3′) (Fasmac Co. Ltd., Kanagawa, Japan) were mixed and annealed in RNA buffer (10 mM Tris-HCl (pH 7.9), 20 mM NaCl, 2 mM MgCl_2_, 0.2 mM DTT) for 2 min at 95 °C. Two microliters (2 µL) of the annealed RNA solution was incubated with 5 µg *Streptococcus pyogenes* Cas9 protein (Fasmac Co.) in RNA buffer for 15 min at 37 °C.

The homology-directed repair (HDR) donor was amplified by PCR using the *aphVIII* paromomycin-resistance gene cassette as a template [32]. Each of the forward (HDR-F) and reverse (HDR-R) primers has a 50-bp homology arm corresponding to the exon 2 and intron 2 regions of the *fap70* genomic sequence, respectively. The PCR products were purified, ethanol-precipitated, and resuspended in 10 mM Tris-HCl (pH 8.0) buffer. The sequences of HDR-F and -R were as follows:

HDR-F: 5′-GCTCGCCAGCGCCACCGTTGACCAGCTGCAGGGGTTTGCAATCGGTCAGAGCAATAGATCCGCTGAGGCTTG-3′

HDR-R: 5′-AGCGGCCGGCGCGCGCACCTTGGGCACCCCCTCCGGCACCTCCGCTGCGGAGCTGGGTACCGCTTCAAATACG-3′

*cc-125* wild type cells were treated with autolysin for 1 h at 25 °C. The treated cells were then heated at 40 °C for 30 min. The cells were washed once with Tris-acetate-phosphate (TAP) media supplemented with 2% sucrose and then centrifuged at 2400 rpm for 2 min. The cell pellet was resuspended in TAP-sucrose media, and 110 µL of the cell suspension was mixed with 10 µL of the RNA–Cas9 complex solution and 2 µg of HDR-donor DNA followed by electroporation using a BTX ECM 630 device (Harvard Apparatus, Holliston, MA, USA) at 350 V, 25 Ω, and 600 µF. The cuvettes were incubated for 1 h at 16 °C, and the cells were diluted with 10 mL TAP-sucrose and incubated for 24 h at 25 °C. Cells were then plated on TAP agar plates containing 10 µg/mL paromomycin (Nacalai Tesque, Kyoto, Japan). Colonies were randomly picked and screened by PCR analysis. About 55% of the picked clones were found to be insert-positive in exon 1 of the *FAP70* gene.

### 2.9. Analysis of Chlamydomonas Flagella

Axonemes of *Chlamydomonas* cells were purified as described [33]. Genomic DNA of FAP70 was subcloned and inserted into the pIC2-N-BCCP plasmid as described [34]. Fluorescence staining of axonemes was conducted as described [35,36]. The swimming velocity of *Chlamydomonas* cells was recorded as described [35]. Waveform of *Chlamydomonas* flagella were analyzed as described [37]. Streptavidin-cytochrome *c* labeling of BCCP-tagged axonemes was carried out as described previously [22,37]. Image acquisition on a cryo-electron microscope was carried out as described [37,38]. Image processing for subtomogram averaging of doublet microtubule (DMT) structures was carried out as described previously [22,23,39,40,41]. The numbers of DMT subtomograms averaged were 1480 for *fap70* and 1448 for *fap70∷FAP70-N-BCCP*. The effective resolutions determined by Fourier Shell Correlation with a cutoff value of 0.5 were ~4 nm (Figure S2B). Surface renderings were generated using UCSF Chimera [42]. The electron microscopy map of *fap70∷FAP70-N-BCCP* is available at the EM Data Bank (www.emdatabank.org) under the accession number EMD-9618. To identify statistically significant differences, we applied Student’s *t*-test to compare wild-type and streptavidin-labeled axonemes as described previously [22,39]. Chromatographic separation of axonemal dyneins was conducted as described [43].

### 2.10. Statistical Analysis

Differences between two groups were compared using a two-tailed *t* test. Results were considered significant at *p* < 0.05.

## 3. Results

### 3.1. The Expression of CFAP70 in Mouse is Associated with Motile Cilia and Flagella

To seek novel cilia-related genes, we compared the transcriptomes of cultured mouse ependyma (having hundreds of cilia per cell) and choroid plexus epithelial cells (having dozens of cilia per cell) [28]. Among the list of genes, *Cfap70* (or *Ttc18*) caught our attention as the expression levels appeared to be correlated with the ciliary number, just like *Ttc21a* (*Ift139a*) as well as *Ttc25*, which was recently reported as a novel ciliopathy gene [44] (Supplementary Table S1).

**Figure 1:**
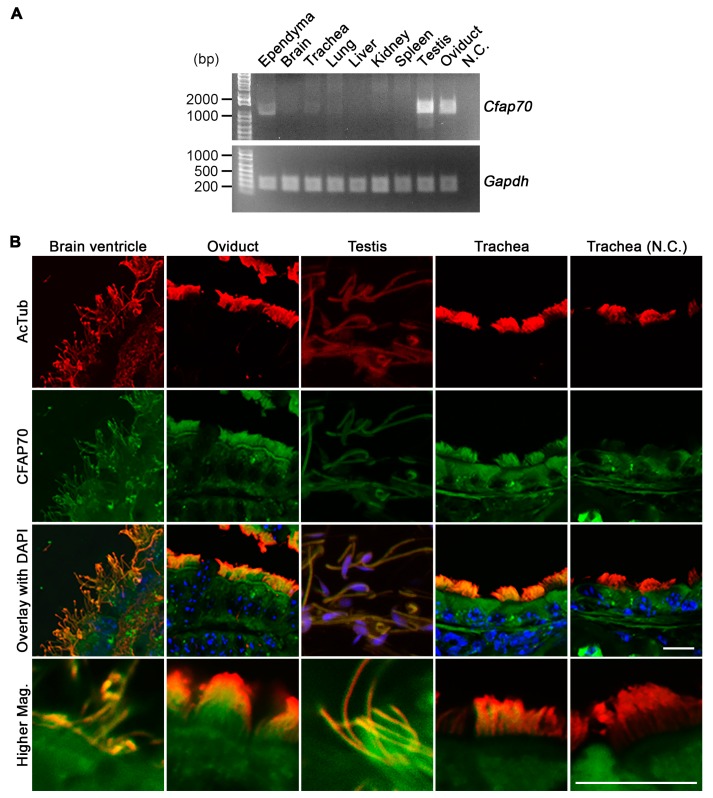
Detection of *Cfap70* mRNA and protein in mouse. (**A**) Semi-quantitative RT-PCR analysis of various cells and tissues. To the left of the gel, the positions and sizes (bp) of DNA ladders are indicated. (**B**) Confocal microscopy of tissue sections immunostained for CFAP70 (green) and acetylated α-tubulin (AcTub, red) along with DAPI (blue). As a negative control (N.C.), a tracheal section was immunostained using mouse anti-AcTub and rabbit normal IgG. All of the CFAP70-positive epithelia and spermatocytes have motile 9 + 2 cilia and flagella. Bars, 10 µm.

To investigate the function of CFAP70, the mRNA levels in various mouse tissues and cells were assessed by reverse transcription-polymerase chain reaction (RT-PCR). Total RNA was extracted from whole brain, trachea, lung, liver, kidney, spleen, testis, and oviduct, as well as the ependymal culture. Two micrograms of each RNA sample were then used for reverse transcription. From the following semi-quantitative PCR analysis, *Cfap70* mRNA was detected in cultured ependyma, trachea, lung, testis, and oviduct, but not in whole brain, liver, kidney, and spleen (Figure 1A). As all of these *Cfap70*-positive cells and tissues are rich in cells that have motile cilia and flagella, the ciliary localization of CFAP70 protein in these tissues was investigated by immunostaining. The subsequent fluorescence microscopy demonstrated that CFAP70 localizes on the epithelial cilia and sperm flagella (Figure 1B). The specificity of the immunoreactive signal was validated by a control experiment using normal rabbit IgG instead of the anti-CFAP70 antibody. CFAP70 was undetected in kidney sections by immunostaining, consistent with the RT-PCR data (data not shown). These data demonstrate that mouse CFAP70 does associate with motile cilia and flagella as in the *Chlamydomonas* homolog FAP70 [24].

### 3.2. CFAP70 Binds Tightly to the Axoneme of Motile Cilia

**Figure 2:**
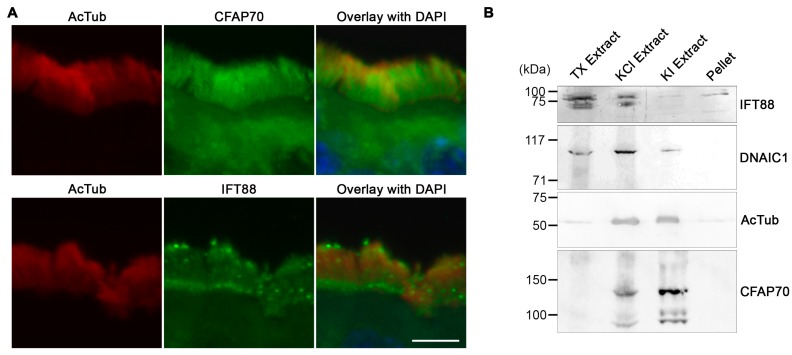
Comparison of the intra-ciliary distribution between CFAP70 and IFT88. (**A**) Confocal microscopy of mouse tracheal tissue immunostained for CFAP70 (top panels) and IFT88 (bottom panels). Bar, 5 µm. (**B**) Isolated swine tracheal cilia were extracted sequentially with 0.1% Triton X-100, 0.6 M KCl, and 0.6 M KI. The resulting supernatants (TX, KCl, and KI, respectively) and final pellet were analyzed by Western blot using antibodies against the indicated ciliary proteins. To the left of the gel, the positions and sizes (kDa) of molecular weight standards are indicated.

Based on the fact that many cilia-associated, TPR-domain-containing proteins that have been characterized so far are components of the IFT complex [45], the intra-ciliary localization of CFAP70 was investigated with particular interest in its potential association with the IFT complex. Although the resolution of the images was insufficient to make a solid conclusion, immunostaining of mouse tracheal cilia implied that CFAP70 and IFT88 might be distributed differently, since the IFT88 signal appeared more in particles in and at the base of the cilia (Figure 2A). These distinct staining patterns suggest that CFAP70 is unlikely to be a component of the IFT complex.

To consolidate this observation, a biochemical fractionation of swine tracheal cilia was performed. A crude preparation of motile cilia liberated from the epithelium by vortex was extracted stepwise with 0.1% Triton X-100, 0.6 M potassium chloride, and 0.5 M potassium iodide. It has been shown previously that, while the initial detergent treatment solubilizes the ciliary membrane and liberates the matrix proteins, including the IFT complex, the following KCl treatment extracts most of the dynein arm complexes, and the final KI treatment dissolves the remaining axonemal proteins in *Chlamydomonas* flagella [24]. As expected, Western blot analysis of the stepwise extracts demonstrated that IFT88 was dissolved readily by the initial Triton X-100 treatment, and the majority of DNAIC1 (axonemal dynein intermediate chain 1) was found in the second KCl fraction, whereas a significant amount of the acetylated α-tubulin remained insoluble until the final KI treatment (Figure 2B). The distribution of CFAP70 was distinct from that of IFT88 in that the protein was not solubilized by the detergent extraction and was dissociated by the KCl and KI extractions (Figure 2B). Altogether, these data demonstrated that CFAP70 is not a component of IFT and that it associates tightly to the ciliary axoneme. These biochemical properties of CFAP70 were comparable with the report on *Chlamydomonas* FAP70 by Pazour et al., in which a stepwise extraction and the following proteomic analysis of the flagella demonstrated that FAP70 was enriched in the KCl extract [24].

### 3.3. The Conserved TPR Domains of CFAP70 Are Unnecessary for Ciliary Localization

**Figure 3:**
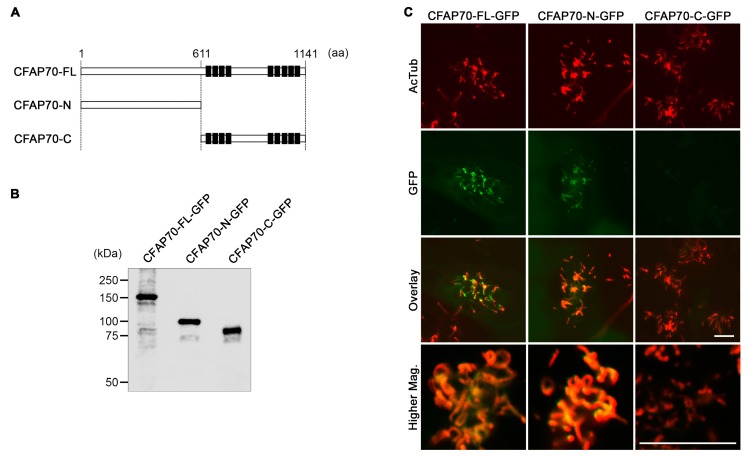
Changes in the ciliary localization of CFAP70 fragments in primary culture of mouse ependyma. (**A**) Schematic diagrams of mouse CFAP70 protein and its deletion constructs made in this study. The 1141-amino-acid protein has clusters of tetratricopeptide repeat (TPR) domains (indicated by filled boxes) at the C-terminal half. (**B**) Western blot analysis for validation of the expression of recombinant CFAP70 proteins fused with a GFP tag at the C-terminus. 293T cells were transfected with the lentiviral expression plasmids and the whole cell lysates were analyzed. The calculated molecular weights are as follows: CFAP70-FL-GFP, 155 kDa; CFAP70-N-GFP, 95 kDa; CFAP70-C-GFP, 87 kDa. To the left of the gel, the positions and sizes (kDa) of molecular weight standards are indicated. (**C**) Confocal microscopy of cultured ependyma transduced with lentiviral particles carrying the above constructs. The cells were immunostained for AcTub (red) and GFP (green). Bars, 10 µm.

CFAP70 has nine TPR domains that cluster at the C-terminal half (Figure 3A). To investigate if the TPR domains are necessary for ciliary localization, full-length *Cfap70* cDNA and the fragments encoding either the N- or C-terminal half were subcloned into a lentiviral GFP expression vector (Figure 3A,B). The sequence of full-length *Cfap70* cDNA cloned from cultured mouse ependyma was identical to NM_029698 in the NCBI database. These constructs were packaged into lentiviral particles and transduced into cultured ependyma. The transduced cells were then immunostained using anti-GFP and anti-AcTub antibodies for fluorescence microscopy. While a significant amount of GFP signals was present in the cell body, the full-length construct (CFAP70-FL-GFP) was targeted to cilia, and its colocalization with AcTub was readily detected (Figure 3C). The N-terminal fragment lacking TPR domains (CFAP70-N-GFP) was also detected in cilia, though the signal was weaker than that of the full-length construct. By contrast, the C-terminal fragment with TPR domains (CFAP70-C-GFP) was undetected in cilia and distributed broadly in the cytoplasm. These data demonstrate that the conserved TPR domains are dispensable for ciliary targeting and that the N-terminal half is more important than the C-terminal half for entry to the cilia and/or binding to the axoneme.

### 3.4. Knockdown of CFAP70 in Mouse Ependyma Causes a Reduction in Ciliary Motility

To gain insight into the function of CFAP70, the gene expression in cultured mouse ependyma was knocked down by lentiviral transduction of the shRNA expression cassette. For this, the knockdown efficiency of five different pLKO1.0 puro lentiviral shRNA constructs against *Cfap70* was evaluated in 293FT cells by co-transfecting them with the CFAP70-FL-GFP expression plasmid to select the one with the highest efficiency (data not shown).

**Figure 4:**
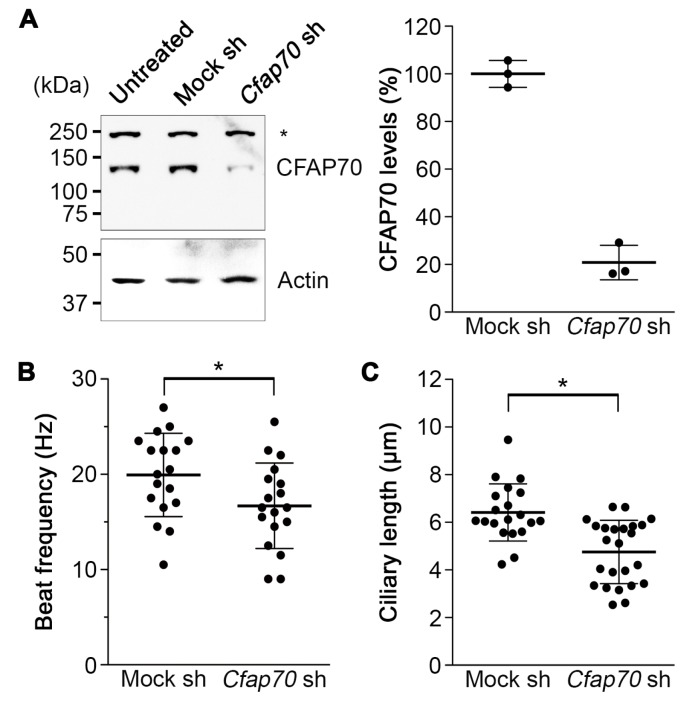
Knockdown of *Cfap70* in cultured mouse ependyma. (**A**) Western blot analysis to assess the knockdown efficiency of lentiviral shRNA in cultured mouse ependyma. Left, whole cell lysates of ependyma treated with viral particles carrying mock or *Cfap70* shRNA were analyzed. The lysate of untreated ependyma was also loaded as a control. To the left of the gel, the positions and sizes (kDa) of molecular weight standards are indicated. The band of endogenous CFAP70 with a calculated size of 128 kDa, but a non-specific band of ~250 kDa (asterisk), was diminished in the sample treated with Cfap70 shRNA. The immunoblot for pan actin shows equal loading. Right, quantification of CFAP70 protein levels. Data are presented as the mean ± SD (*n* = 3). (**B**) Cilia beat frequency was measured based on high-speed video microscopy data. * *p* < 0.05 versus mock-treated control. Data are presented as the mean ± SD (*n* = 18). (**C**) Ciliary length was measured based on the z-stack images of immunostaining for AcTub. * *p* < 0.05 versus mock-treated control. Data are presented as the mean ± SD (*n* = 20 for mock sh, *n* = 24 for *Cfap70* sh).

Cultured mouse ependyma were transduced with the virus carrying the selected clone at a multiplicity of infection of 1.0, and untransduced cells were eliminated using 1.0 µg/mL puromycin. As a negative control, an empty vector expressing mock shRNA was used. The knockdown efficiency was validated by Western blot, which showed ~80% reduction in endogenous CFAP70 protein levels (Figure 4A).

Having confirmed the high knockdown efficiency in the primary culture, the effects on the motile cilia were investigated. The analysis of ciliary motility by video microscopy and the following immunostaining for AcTub demonstrated a reduction in cilia beat frequency and ciliary length (Figure 4B,C). These data demonstrate that CFAP70 plays a role in the regulation of ciliary motility.

### 3.5. Chlamydomonas Homolog of CFAP70, FAP70, Is Important for Proper ODA Function and Localizes at the Base of ODA

**Figure 5:**
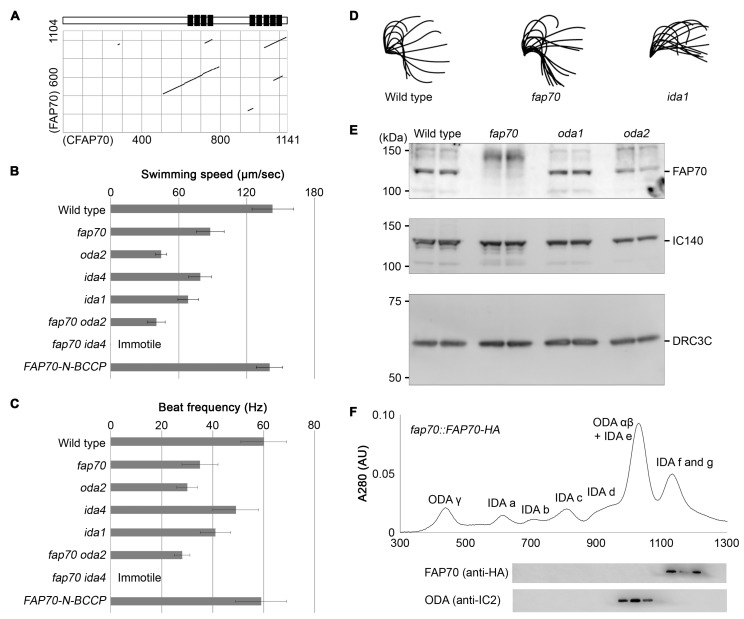
Characterization of a *Chlamydomonas reinhardtii* mutant lacking FAP70. (**A**) Analysis of the sequence similarity between mouse CFAP70 and *Chlamydomonas* FAP70 proteins. The dot plot was generated by a BLASTP program [46,47], where CFAP70 (1141 amino acids) is represented on the X-axis and FAP70 (1104 amino acids) is represented on the Y-axis. Matched sequence alignments are shown as lines. Shown on the top of the plot is the CFAP70 schematic diagram shown in Figure 3A. (**B**,**C**) The swimming speed (**B**) and flagellar beat frequency (**C**) of *Chlamydomonas.* Wild-type, three single mutants (*fap70*, *oda2*, *ida4*, and *ida1*), two double mutants (*fap70 oda2* and *fap70 ida4*), and the *fap70* mutant expressing FAP70-N-BCCP (*FAP70-N-BCCP*) were analyzed. Data are presented as the mean ± SD (*n* = 20). (**D**) Representative tracings of the flagellar beating form. (**E**) Western blot analysis of FAP70, IC140 (inner dynein arm), and DRC3C (dynein regulatory complex) protein levels in wild-type and three different mutants (*fap70*, *oda1*, and *oda2*). Twenty micrograms of axonemal protein lysate were loaded on each lane. To the left of the gel, the positions and sizes (kDa) of molecular weight standards are indicated. (**F**) Top, a 0.6 M KCl extract from *fap70::FAP70-N-BCCP-HA* axonemes was separated by MonoQ anion-exchange chromatography column. The protein peaks of known dynein arm complexes are labeled. Bottom, the MonoQ fractions were separated by SDS-PAGE and FAP70 and outer dynein arms (ODAs) were detected using anti-HA and anti-IC2 antibodies, respectively.

To investigate the molecular function and axonemal localization of CFAP70 in more detail, a *Chlamydomonas reinhardtii* mutant lacking the *CFAP70* homolog, *FAP70*, was analyzed. Dot plot analysis of mouse CFAP70 and *Chlamydomonas* FAP70 amino acid sequences (NP_083974 and XP_001692552, respectively) indicated that they share a common overall structure, with the TPR domain clusters conserved at the C-terminal half (Figure 5A). The prediction of the protein secondary structure using the psipred server program (bioinf.cs.ucl.ac.uk) also demonstrated an overall similarity between CFAP70 and FAP70; while the C-terminal regions with the conserved TPR domains were rich in α helices, the N-terminal regions were rich in β sheets (Supplementary Figure S1).

We generated a *FAP70-null* mutant by using CRISPR/Cas9 [31,48,49,50]. The knockout clones were selected by paromomycin and subsequent genomic PCR using a primer pair flanking the target locus (Supplementary Figure S2). The isolated *fap70* mutants exhibited a typical ODA phenotype characterized by intact waveforms and reductions in swimming speed due to a low flagellar beat frequency (Figure 5B–D; Supplementary Videos S1 and S2). Several clones were isolated by two independent CRISPR/Cas9 knockout experiments, and they shared the same phenotype. The lack of FAP70 protein in the mutant clone was confirmed by Western blot of the flagellar axoneme (Figure 5E). The axoneme of *oda1* and *oda2* mutants, lacking the outer dynein arm components, had FAP70 at levels comparable to that of wild-type. The loss of FAP70 had no effect on the protein levels of IC140 [51] and DRC3 [34], which are the components of the inner dynein arm and the nexin–dynein regulatory complex (N-DRC), respectively (Figure 5E). The loss of FAP70 also had no effect on flagellar length (data not shown). The defect in flagellar motility was rescued by FAP70 expression (Figure 5B,C). In addition, when the *fap70* mutant was crossed with *oda* or *ida* mutants to generate double mutants, the *fap70* × *oda2* mutant had an ODA phenotype similar to the parental single mutants, whereas the *fap70* × *ida4* mutant exhibited a combination of ODA and IDA phenotypes and completely lost the ability to swim (Figure 5B,C). These data demonstrated that loss of FAP70 causes a defect in ODA activity.

To investigate if FAP70 is a component of ODA, the purified flagellar axonemes from a rescued *fap70* mutant (*fap70::FAP70-N-BCCP-HA*; Supplementary Figure S3A) were demembranated and extracted with 0.6 M KCl, and the dynein arms in the resulting extract were separated by a MonoQ column. The Western blot demonstrated that FAP70-HA was absent in the fractions containing ODA α and β and was eluted along with IDA *f* and *g* (Figure 5F). These results demonstrated that FAP70 is not a component of the ODA complexes.

**Figure 6:**
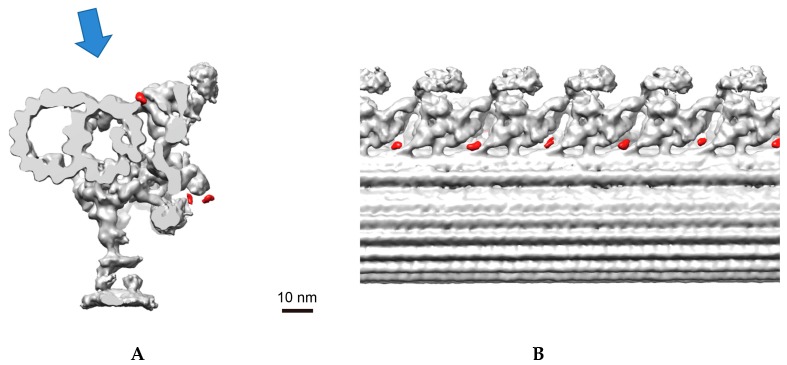
Analysis of the localization of FAP70 protein by cryo-electron tomography. (**A**) A cross-sectional view of one out of nine doublet microtubules of the axoneme. The flagella of the *fap70* mutant rescued by FAP70-N-BCCP were purified, and the axoneme was labeled with streptavidin and biotinylated cytochrome *c* to increase the density of the BCCP tag. The resulting three-dimensional (3D) structure of the subject by cryo-electron tomography was compared with the wild-type reference data, and the tag densities found only in the subject are labeled in red. The arrow indicates the direction of the views in **B**. (**B**) Longitudinal view. The tag densities were located at the base of the ODAs.

Having confirmed the ODA functional defect, cryo-electron tomography of the flagellar axoneme was performed to investigate the three-dimensional (3D) ultrastructure. However, no obvious changes in the *fap70* axoneme were observed (Supplementary Figure S3B,C). Next, when a full-length FAP70 fused with a biotin carboxyl carrier protein (BCCP) tag at the N-terminus (FAP70-N-BCCP) was expressed in the mutant clone and its localization on the flagellar axoneme was investigated, the densities of the streptavidin label were found at the base of the ODA (Figure 6). There were also significant signals of the labels on the N-DRC (Supplementary Figure S3D). As the *fap70* mutant did not show any functional or structural defects in the N-DRC, we cannot identify the functional meaning of the localization of FAP70 on the N-DRC. Altogether, these data demonstrate that FAP70 associates with the axoneme, like mammalian CFAP70, localizes at the base of the ODA, and regulates its function.

## 4. Discussion

Despite the fact that *Cfap70* and its homologous genes are conserved among a wide range of species, functional analyses have been sparse. So far, two groups have reported the phenotype of zebrafish morphants of the *Cfap70* homolog, *ttc18*. Xu et al. demonstrated a systematic screening to identify novel cilia-related TTC genes [52]. In their article, *Cfap70* was found by their initial screening to be one of the upregulated TTC genes in differentiating mouse tracheal epithelial cells. In their second screening, knockdown of *ttc18* in zebrafish resulted in only a minor body-curving phenotype, a patterning defect observed frequently in ciliopathy model zebrafish lines [53], and was not investigated further. In another report by Dam et al., large datasets of genomics, proteomics, transcriptomic, and evolutionary data were integrated to make a list of novel candidate ciliopathy genes [54]. In the course of the validation process, *ttc18* morphants were noted with reduced ciliary number as well as ciliary length in Kupffer’s vesicles and enlarged pronephric ducts and curved bodies. While the specificity and efficiency of morpholinos have been the major concern [55], these reports appear to be consistent with our data in that the expression of CFAP70 is associated with motile cilia and flagella and is important for ciliary motility.

Our data on *Chlamydomonas* FAP70 demonstrated that the protein is a novel regulator of ODA (Figure 5). FAP70 is dissociated from the well-defined ODA complexes upon treatment with 0.6 M KCl, which is unique since all the causative genes of the ODA defect identified so far encode stable components of ODA complexes in 0.6 M KCl. In agreement with this biochemical property, the lack of FAP70 did not affect the ODA ultrastructure as demonstrated by cryo-electron tomography (Figure S2). Therefore, rather than contributing to the structural integrity of ODA, FAP70 may be involved in the coordination of ODA activity in response to changes in the surrounding mechanical and/or chemical conditions to produce proper ciliary motility.

Whereas the primary amino acid sequence suggested the importance of the conserved TPR domains at the C-terminus, our data demonstrated that it is the N-terminal end of FAP70 that localizes stably at the base of the ODA (Figure 6). By contrast, when the BCCP tag was fused to the C-terminus, the location on the axoneme was undetermined by cryo-electron tomography, suggesting a flexible nature of the end (T. Oda, personal communication). These observations are also consistent with the data on CFAP70 in mouse ependyma demonstrating the importance of the N-terminus for ciliary localization (Figure 3). Based on the fact that TPR domains are known to mediate protein–protein interactions, the molecular function of CFAP70 and FAP70 might be to anchor a certain protein in close proximity to the ODA; while the N-terminal region is fixed at the base of the ODA, the C-terminal region may define the binding partner that regulates ODA function. As possible binding partners, various kinds of protein that regulate ciliary motility but whose locations on the axoneme are unknown, such as protein phosphatases [56] and calcium-binding proteins [57], can be considered.

While the present study has demonstrated high similarities between CFAP70 and FAP70, some differences did exist. In the stepwise extraction of swine tracheal cilia, a large portion of CFAP70 was not released until treated with 0.6 M KI (Figure 2B), while FAP70 was extracted readily by KCl [24]. This could be simply due to the differences in the binding affinity to the axoneme, but it might imply that CFAP70 has other binding partners, such as radial spokes. In addition, when CFAP70 was knocked down in mouse ependyma, the ciliary length was reduced (Figure 4C), which is difficult to explain from the viewpoint of ODA regulation. Further investigations will clarify the mechanism by which CFAP70 regulates ODA function and ciliary length in mammals.

## Supplementary Materials

The following are available online at http://www.mdpi.com/2073-4409/7/9/124/s1, Figure S1: Prediction of the protein secondary structure using the psipred server program (bioinf.cs.ucl.ac.uk), Figure S2: Genome editing of *Chlamydomonas FAP70* by CRISPR/Cas9, Figure S3: Investigation of the localization of FAP70 in *Chlamydomonas* flagella, Table S1: Comparison of the expression levels of various *Ttc* genes between cultured mouse ependyma (mEPD) and choroid plexus epithelial cells (mCPE), Video S1: Swimming activity of wild-type *e*, Video S2: Swimming activity of *fap70* mutant.
