# Supplementary material for: CFAP70 Is a Novel Axoneme-Binding Protein That Localizes at the Base of the Outer Dynein Arm and Regulates Ciliary Motility
Source: Cells. 2018 Aug 29;7(9):124. doi: 10.3390/cells7090124 (PMC6162463; doi:10.3390/cells7090124)
Supplement: Supplementary file 1 [file cells-07-00124-s001.zip › cells-345533-supplementary/cells-345533-supplementary.pdf]

cells

MDPI

Article

CFAP70 is a novel axoneme-binding protein that localizes at the base of the outer dynein arm and regulates ciliary motility

Noritoshi Shamoto [1,†], Keishi Narita [1,*,†], Tomohiro Kubo [2], Toshiyuki Oda [2] and Sen Takeda [1,*]

Supplemental Methods

Rabbit polyclonal antibody production and purification

Rabbit polyclonal antibodies against *Chlamydomonas reinhardtii* FAP70 were produced using a synthetic oligopeptide, SEGGVYQYSVVKHFC, corresponding to the position 68-81 and the terminal cysteine for chemical crosslink with keyhole limpet hemocyanin. The custom peptide conjugated with the carrier protein was obtained from Cosmo Bio (Tokyo, Japan), and was used for immunization of rabbits at EveBioscience (Wakayama, Japan) to obtain antisera.

For affinity purification of anti-FAP70 antibodies, a GeneArt Strings DNA fragment, corresponding to the coding sequence of FAP70 in exons 2 and 3 (the codon usage was optimized for expression in *E. coli*), fused with poly-histidine tag at the 3′ end and flanked by BamHI and SalI restriction sites, was synthesized by Thermo Fisher Scientific (Waltham, MA), and ligated into pGEX6P-1 (GE Healthcare Japan, Tokyo, Japan). The resulting construct was introduced into *E. coli* BL21(DE3) and used for protein overexpression and subsequent purification using His60 Ni Gravity Columns (Takara Bio, Shiga, Japan) in the presence of 6 M guanidine hydrochloride. The purified recombinant FAP70 fragment was resolved by SDS-PAGE and transferred on a PVDF membrane, and the membrane strip was used for affinity purification of the antibodies.

Reverse transcription-polymerase chain reaction (RT-PCR)

The sequences of the custom oligonucleotide primers (Life Technologies) used for PCR are as follows: *Cfap70* Fwd, 5′-TGTGAGTGACTACCACACGC-3′; *Cfap70* Rev, 5′-ACAGGAGGCTGGGCAATAAC-3′; *Gapdh* Fwd, 5′-AACTTTGGCATTGTGGAAGG-3′; *Gapdh* Rev, 5′- ACACATTGGGGGTAGGAACA-3′. The annealing temperatures for both primer pairs were set to 60°C.

Construction of lentiviral plasmid expressing full-length and truncated CFAP70

The full-length *Cfap70* cDNA was amplified from the cDNA library of cultured mouse ependyma, using KAPA HiFi DNA polymerase. The sequences of the custom oligonucleotide primers are: *Cfap70* NheI Fwd, 5′-GCCGCCGCTAGCATGGACCAGACGTCAAGCACAACG-3′; *Cfap70* AgeI Rev, 5′-GAAGAAACCGGTCCGAAAGATGGATTTCCAAAACCAACCATTTCCTG -3′. For the cloning of *Cfap70* cDNA fragments encoding the N- and C-terminal halves, the following primers were used in combination with the above primers: *Cfap70* 1830 AgeI Rev, 5′-GAAGAAACCGGTCCTTGATCTACCAAGAACACATACAGCTCACTGATAAA-3′; *Cfap70* 1831 NheI Fwd, 5′-GCCGCCGCTAGCATGCATGTGGCCCTGAACCAGG-3′. The annealing temperature for these primer pairs was set to 65°C. These PCR products were ligated into the NhiI-AgeI site of CS-CDF-CG-PRE.

Screening of lentiviral shRNA clones

For the knockdown of *Cfap70* expression in cultured mouse ependyma, the following pLKO1.puro lentiviral shRNA expression constructs targeting different sites of *Cfap70* mRNA from each other were obtained from Dharmacon (Lafayette, CO, USA): TRCN0000346337, TRCN0000346338, TRCN0000346340, TRCN0000346341, and TRCN0000346412. These plasmids were tested for their knockdown efficiency in 293T cells by co-transfecting each clone with the CFAP70-FL-GFP expression construct, followed by fluorescence microscopy and western blot.

Supplementary Table S1. Comparison of the expression levels of various *Ttc* genes between cultured mouse ependyma (mEPD) and choroid plexus epithelial cells (mCPE).

Gene_Symbol	global normalization			Fold Change	
	Cy5_mEPD	Cy3_mCPE	LOG_2 [ratio(Cy5/Cy3)]	up	down
Ttc1	490.7	785.3	-0.68		
Ttc5	186.0	114.6	0.70		
Ttc7	24.0	97.6	-2.02		**
Ttc7b	79.5	170.6	-1.10		*
Ttc8	756.4	224.0	1.76	*	
Ttc9	544.4	178.2	1.61	*	
Ttc9b	33.4	n.d.		–	
Ttc12	71.8	99.2	-0.47		
Ttc13	493.1	1012.2	-1.04		*
Ttc14	71.6	104.1	-0.54		
**Ttc18**	528.4	26.6	4.31	***	
**Ttc21a** [1]	122.8	10.9	3.49	***	
**Ttc25** [2]	644.1	62.8	3.36	***	
Ttc27	275.1	116.1	1.25	*	
Ttc28	470.1	171.5	1.45	*	
Ttc28	16.7	5.6	1.57	*	
Ttc29	270.4	n.d.		–	
Ttc33	346.3	474.9	-0.46		
Ttc35	1857.4	3186.4	-0.78		

n.d., not determined. [1] Also known as Ift139a. [2] Am J Hum Genet, 99(2):460-9 (2016)

CFAP70

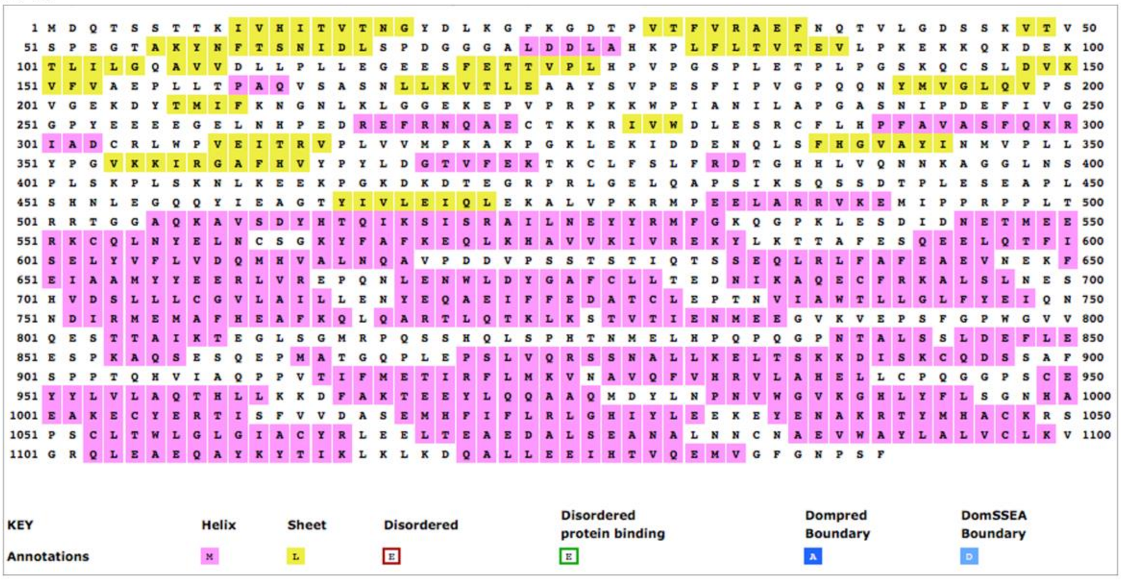

FAP70

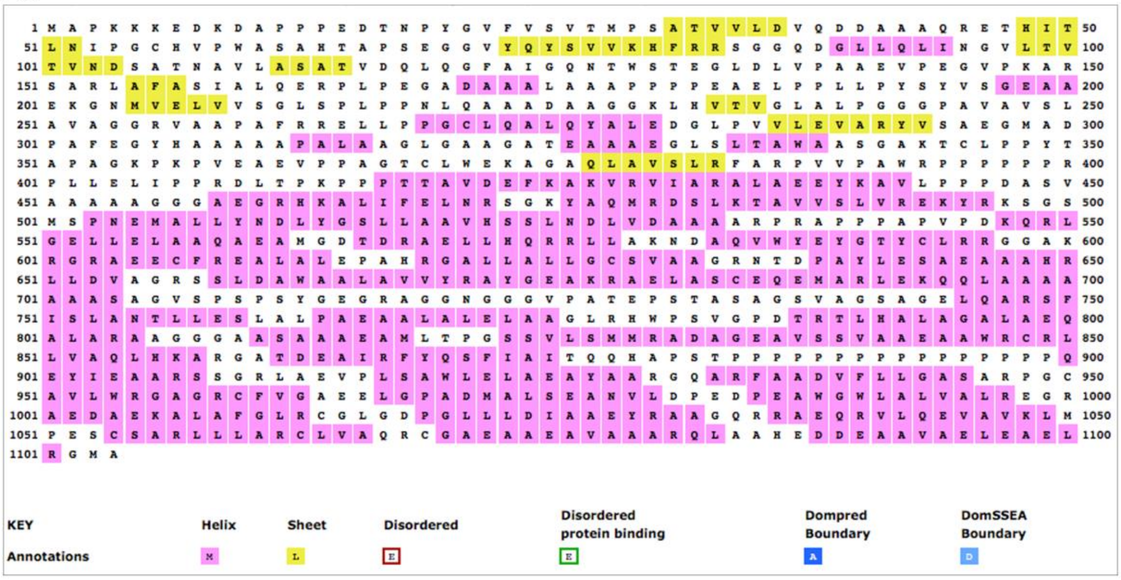

Figure S1. Prediction of the protein secondary structure using the psipred server program (bioinf.cs.ucl.ac.uk).

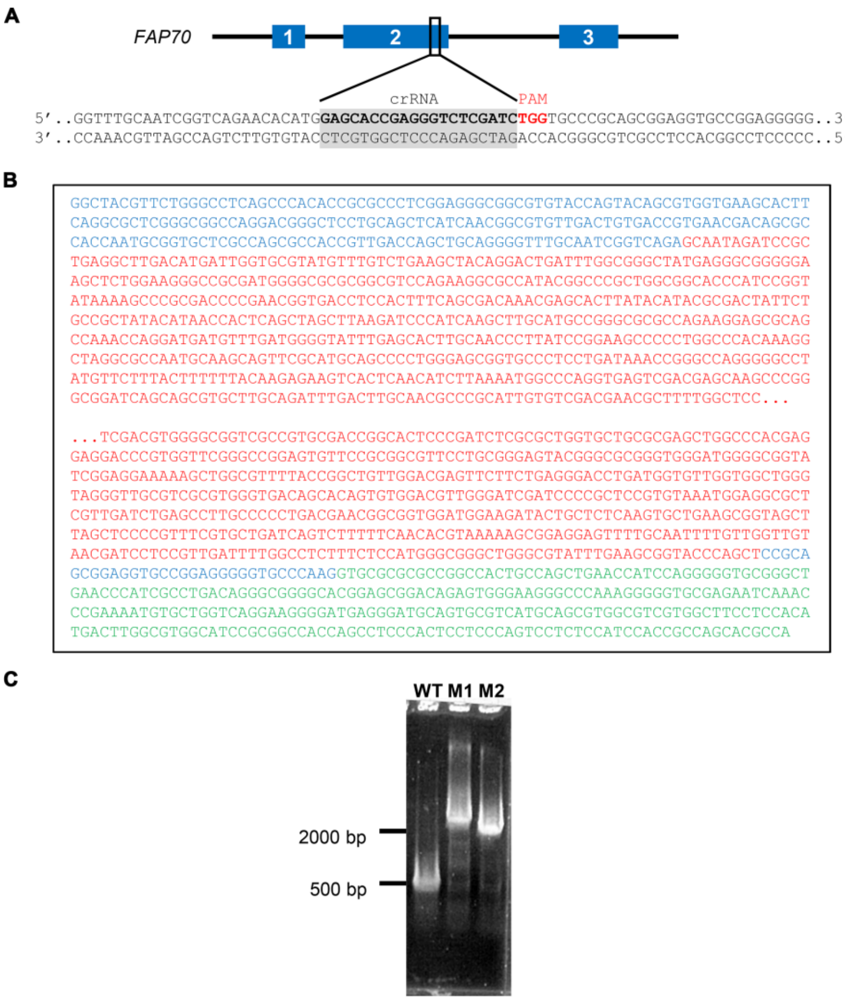

Figure S2. Genome editing of *Chlamydomonas FAP70* by CRISPR/Cas9. (**A**) Schematic diagram of *FAP70* exons 1-3. The position of crRNA target sequence and the adjacent protospacer-adjacent motif (PAM) are indicated. (**B**) Partial sequences of a *fap70* mutant genome showing the insertion of an *APHVIII* expression cassette (red) at the exon 2 (blue). Shown in green is the intron 2. (**C**) A representative image of agarose gel electrophoresis to screen *fap70* mutants by genomic PCR. The mutants (M1 and M2) had ~1.5 kb insert of paromomycin resistance gene expression cassette at the targeted site. WT, wild type.

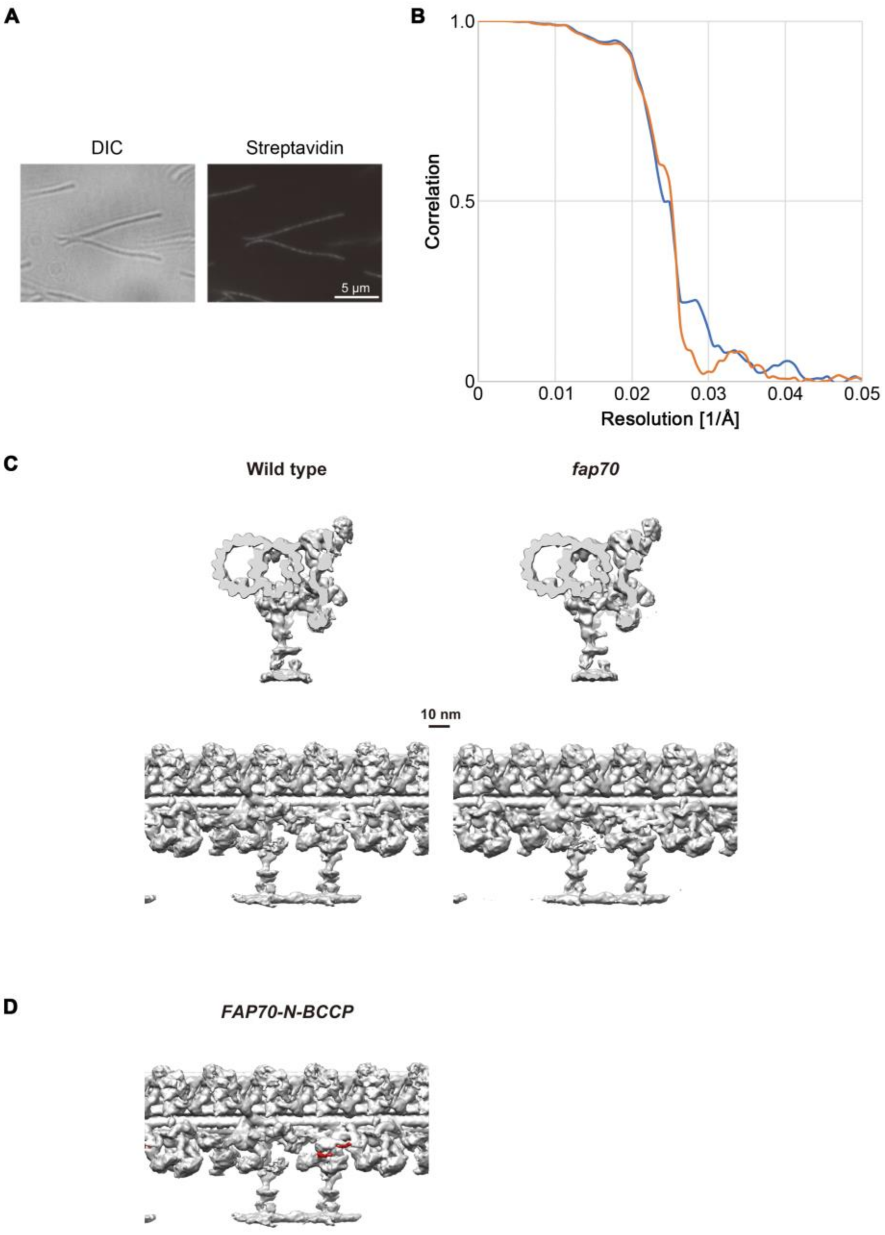

Figure S3. Investigation of the localization of FAP70 in *Chlamydomonas* flagella. (**A**) Purified flagellar axonemes from a rescued *fap70* mutant by FAP70-N-BCCP-HA expression. The structural intactness was confirmed by differential interference contrast (DIC) microscopy, and the recombinant FAP70 labeled with alexa546-conjugated streptavidin was detected by fluorescence microscopy. (**B**) The Fourier shell correlation curves for the reconstruction of *fap70* (blue) and a rescued *fap70* (*fap70*∷*FAP70-N-BCCP*; red) axonemes. The effective resolutions with a cutoff value of 0.5 were ~4 nm. (**C**) A cross-sectional and a longitudinal views of the doublet microtubules from wild type (left) and *fap70* (right) flagella, showing no apparent differences between each other. (**D**) A longitudinal view of the doublet microtubules from a *fap70* mutant rescued by FAP70-N-BCCP. The tag densities (red) were present at the N-DRC, in addition to the base of the ODAs (Fig. 6).
